# Long Non-Coding RNA Neighbor of BRCA1 Gene 2: A Crucial Regulator in Cancer Biology

**DOI:** 10.3389/fonc.2021.783526

**Published:** 2021-12-02

**Authors:** Ting Wang, Zhaosheng Li, Liujia Yan, Feng Yan, Han Shen, Xinyu Tian

**Affiliations:** ^1^ Department of Laboratory Medicine, Jiangsu Cancer Hospital & Jiangsu Institute of Cancer Research & The Affiliated Cancer Hospital of Nanjing Medical University, Nanjing, China; ^2^ Department of Laboratory Medicine, Nanjing Drum Tower Hospital, Nanjing University Medical School, Nanjing, China

**Keywords:** long non-coding RNA, neighbor of BRCA1 gene 2, cancer, metabolism, epithelial-mesenchymal transition, autophagy

## Abstract

Long non-coding RNAs (lncRNAs) are involved in fundamental biochemical and cellular processes. The neighbor of BRCA1 gene 2 (*NBR2*) is a long intergenic non-coding RNA (lincRNA) whose gene locus is adjacent to the tumor suppressor gene breast cancer susceptibility gene 1 (*BRCA1*). In human cancers, *NBR2* expression is dysregulated and correlates with clinical outcomes. Moreover, *NBR2* is crucial for glucose metabolism and affects the proliferation, survival, metastasis, and therapeutic resistance in different types of cancer. Here, we review the precise molecular mechanisms underlying *NBR2*-induced changes in cancer. In addition, the potential application of *NBR2* in the diagnosis and treatment of cancer is also discussed, as well as the challenges of exploiting *NBR2* for cancer intervention.

## Introduction

Presently, it is believed that nearly 87.3% of the human genome is actively transcribed, but <3% of the genome encodes functional proteins (1, 2). Transcripts devoid of protein-coding capacity are termed as non-coding RNAs (ncRNAs) (3, 4). LncRNAs, which were previously recognized as “transcription noise”, represent a class of ncRNAs consisting of >200 nucleotides (5–7). To date, more than 10,000 manually annotated lncRNA genes that produce more than 15,000 lncRNAs have been identified, and their total number continues to grow rapidly, which is due to advancements in RNA sequencing, epigenomic technologies, and computational prediction techniques (2, 8, 9). Furthermore, the increasing numbers of lncRNAs have drawn increased attention to understanding their roles in biology. The lncRNA family is heterogeneous, and individual members can be classified according to their location, structure, function, and transcription orientation (10–12). For instance, based on their location in reference to protein-coding messenger RNAs (mRNAs), lncRNAs can be classified as antisense lncRNAs, lincRNAs, bi-directional lncRNAs, sense lncRNAs, and intronic lncRNAs (6, 13). Similar to mRNAs, the transcription of most lncRNAs is induced by RNA polymerase II, which is accompanied by 5′-capping, 3′-polyadenylation, and splicing (14–16). Although not translated, lncRNAs are emerging as essential modulators of cellular processes through the regulation of chromatin dynamics, gene expression, and protein function (7, 17–20). Functions of lncRNAs are closely associated with their intracellular distribution. LncRNAs in the cytoplasm can interact with mRNAs and proteins, thereby regulating the translation, degradation, and splicing of mRNAs, and inducing changes in protein activity and stability. LncRNAs can also function as “sponges” of microRNAs (miRNAs) in the cytoplasm, leading to the overexpression of miRNA target molecules. For lncRNAs localized in the nucleus, they can recruit transcription activators/repressors to the target gene promoter, thereby facilitating transcriptional activation/silencing. Nuclear lncRNAs can also decoy transcription factors (TFs), resulting in transcriptional inactivation. Moreover, nuclear lncRNAs can induce epigenetic modifications of target genes to regulate their expression (15, 21–25).

Cancer is a complicated disease that associates with a variety of genetic mutations, epigenetic alterations, and chromosomal translocations/deletions/amplifications (26–30). Numerous lncRNAs have been identified as oncogenes or tumor suppressors in a wide range of solid tumors and hematological malignancies, implying that lncRNAs are master regulators in cancer (11, 31, 32). LncRNAs are involved in the regulation of cancer cell proliferation, survival, invasion, and metastasis, as well as the antitumor immune response (33–40). Furthermore, the dysregulated expression of lncRNAs in cancer is associated with the clinical outcome and prognosis of cancer patients (41–44). Among these lncRNAs, *NBR2* is a newly defined lincRNA whose expression is induced by energy stress in the tumor microenvironment (TME) and participates in cancer development (45). Here, we review the role of lncRNA *NBR2* in cancer biology, as well as emphasize its clinical application.

## Identification of the *NBR2* Gene

**Figure 1:**
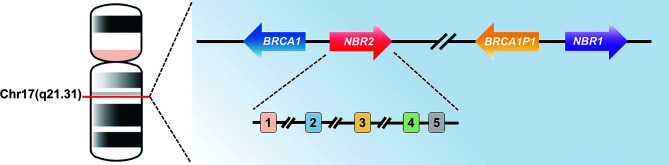
Localization and genomic structure of the human *NBR2* gene. Schematic diagram of the human *NBR2* gene, with arrows indicating the direction of transcription and rectangles representing the exons. Genes are not drawn to scale.

*NBR2* is a non-protein-coding gene on human chromosome 17q21 that spans ~30 kb of genomic DNA and resides adjacent to the *BRCA1* gene (45, 46)*. BRCA1* is a tumor suppressor gene which encodes a nuclear protein that can maintain genome integrity, and germline mutations of the *BRCA1* gene are responsible for most familial cases with breast and ovarian cancer (46–50). To date, more than 100 distinct germline mutations in the coding region of the *BRCA1* gene have been confirmed (51–53). Different from other tumor suppressor genes with both germline and somatic mutations, there are rare somatic mutations in the *BRCA1* gene in breast and/or ovarian cancers. Therefore, *BRCA1* mutations in the coding region are not involved in the development of sporadic cancers, and alternative inactivating mechanisms, such as promoter mutation and DNA hypermethylation, may be involved in the dysregulation of the *BRCA1* gene in sporadic human cancers. Thus, studies aimed at determining the regulation of the *BRCA1* gene are warranted (46, 51, 53). Previous studies, which investigated the 5′ region of the *BRCA1* gene in considerable detail, have revealed the transcription start sites for both *BRCA1* and neighbor of BRCA1 gene 1 (*NBR1*) genes. The genomic region housing the 5′ ends of *BRCA1* and *NBR1* genes is duplicated, as a partial copy of the genomic region encompassing exons 1A, 1B, and 2 of the *BRCA1* gene lies head-to-head with the *NBR1* gene. Meanwhile, a partial copy of the *NBR1* gene, consisting of exons 1A, 1B, and 3, resides adjacent to the transcription start site of the *BRCA1* gene, and this partial copy is identified as a part of the *NBR2* gene that is situated in the genomic region between *BRCA1* and pseudo-*BRCA1* (*BRCA1P1*) genes and lies head-to-head with the *BRCA1* gene (**Figure 1**) (46, 54, 55). Despite high sequence homology at the 5′ ends of *NBR1* and *NBR2* genes, the remaining sequence regions show low homology. The *NBR1* gene has been identified to encode a protein of 966 amino acid residues, which acts as a receptor for the selective autophagosomal degradation of ubiquitinated targets (56, 57). NBR1 is also associated with endosomal membranes by mediating the delivery of certain cargoes. In terms of expression, NBR1 is expressed in all tissues with the highest level in thyroid and testis. There has been little information about the role of NBR1 in cancer. The expression of NBR1 mRNA shows low cancer specificity, and there are weak to moderate NBR1 protein level in cytoplasm of different cancer cells. Decreased NBR1 mRNA level is associated with a poor clinical outcome in patients with clear cell renal carcinoma, indicating NBR1 mRNA level is negatively related with the prognosis of cancer patients. However, other findings revealed the properties of NBR1 in promoting cancer migration and metastasis, and in inducing tumor immune escape by distributing major histocompatibility complex-I (MHC-I) on the cell surface. Therefore, the role of NBR1 in cancer is complicated and needs further investigations (58). Different from the *NBR1* gene, the transcript encoded by the *NBR2* gene is an lncRNA, and its expression has been identified in most examined tissues, including the spleen, thymus, prostate, testis, ovary, small intestine, colon, and peripheral blood leukocytes (46, 59, 60). A previous study has reported that there is an open reading frame (ORF) of 112 amino acid residues in the *NBR2* cDNA that was predicted to encode a ~12-kDa protein. However, a strong Kozak signal has not been observed within this ORF, and the stop codon is located >55 bp upstream of the last splicing site for the putative *NBR2* ORF. Therefore, these findings suggest that the *NBR2* transcript may have been degraded by nonsense-mediated mRNA decay, although it is subjected to protein synthesis (46, 51).

## Regulation of *NBR2* Expression in Cancer

The *NBR2* gene, which is located between *BRCA1* and *BRCA1P1* genes, shows less conservation across species, as it has only been identified in primates but not in other species, including mice. Consistent with the *BRCA1* gene, *NBR2* expression is usually down-regulated in cancer. In a single-strand conformation polymorphism (SSCP) analysis, it was revealed that the *NBR2* gene shows no mutations in both breast and ovarian cancers, suggesting that the involvement of the *NBR2* gene in these cancers is not concerned with gene mutations (46). Sequence analysis of the *NBR2* gene reveals that it contains five exons, in which the last exon is alternatively used and is transcribed in the opposite direction from that of the *BRCA1* gene. The transcription of two distinct *BRCA1* transcripts α and β, in which exon 1A and exon 1B are the first exons, respectively, is achieved by their respective promoters α and β. Promoter α of the *BRCA1* gene is defined to be bi-directional, and *cis*-control elements in the intergenic region of *BRCA1* and *NBR2* genes dominate promoter α activity, as promoter α-induced transcription of both *BRCA1* and *NBR2* genes is regulated by enhancers and silencers located in the region between nucleotide positions 1 and 1357. Moreover, the TF binding sites in the *BRCA1*/*NBR2* promoter have been delineated. Therefore, the expression of *BRCA1* and *NBR2* genes can be affected by different TFs, dominated by *cis*-elements in the intergenic region, and regulated by the availability of these TFs during development and tumorigenesis (54, 61, 62).

**Figure 2:**
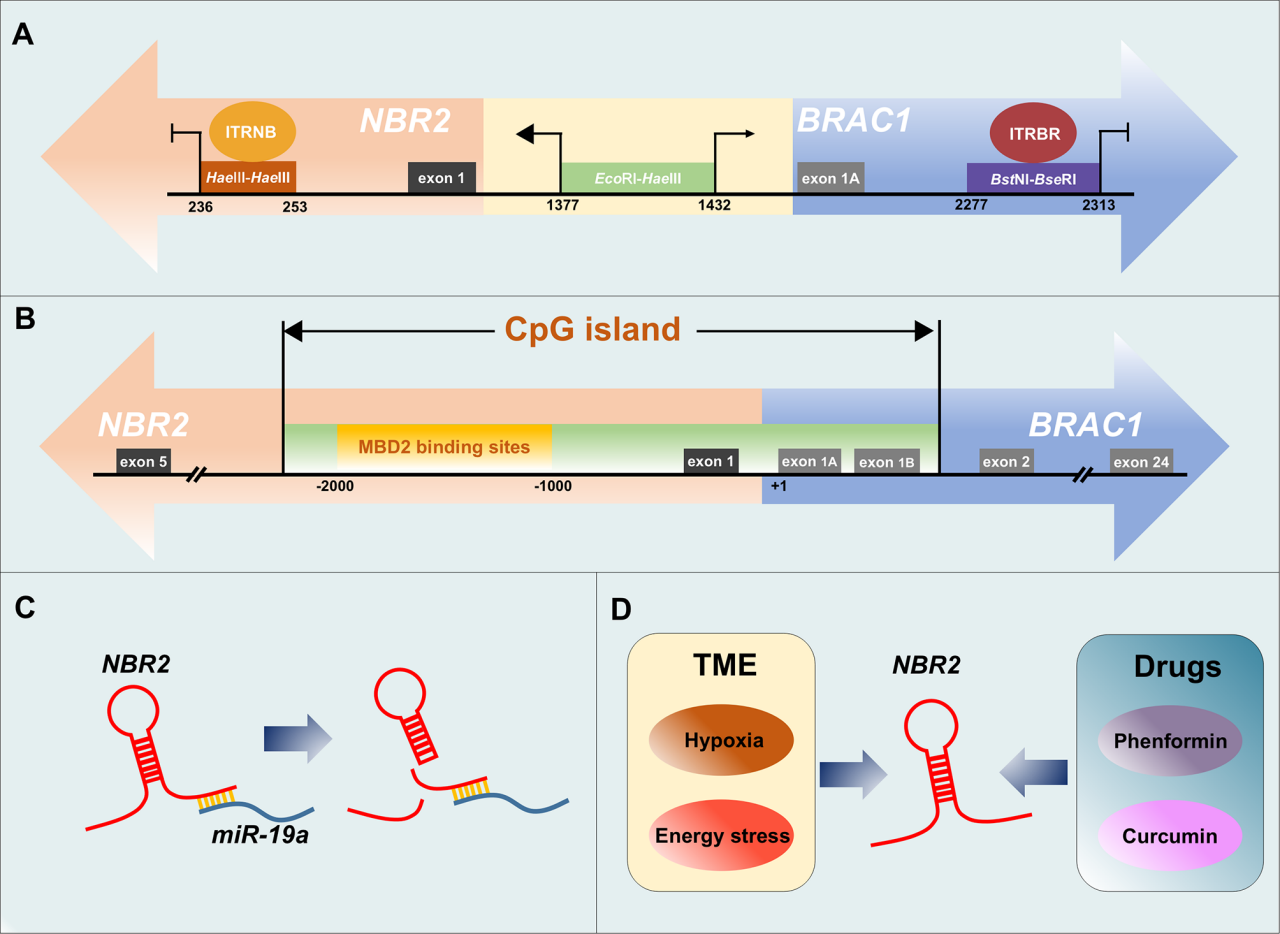
Regulation of *NBR2* expression in cancer. **(A)** Model of the *BRCA1*–*NBR2* bi-directional transcription unit. A minimal 56-bp *Eco*RI–*Hae*III fragment (nucleotides 1377–1432) located between *NBR2* and *BRCA1* genes acts as a bi-directional promoter, and it induces transcription in the *NBR2* gene direction higher than that in the *BRCA1* direction. An 18-bp *Hae*III–*Hae*III repressive element (nucleotides 236–253) that resides 948-bp into the first intron of the *NBR2* gene and is bound by the intronic transcriptional repressor of *NBR2* (ITRNB) functions to turn off transcription only in the *NBR2* direction. The 36-bp *BstN*I–*BseR*I fragment (nucleotides 2277–2313) that resides 575-bp into the first intron of the *BRCA1* gene exhibits non-tissue-specific transcriptional suppressive activity only in the *BRCA1* direction by binding to the intronic transcriptional repressor of *BRCA1* (ITRBR). **(B)** MBD2 leads to the silencing of the *NBR2* gene by binding specifically to the methylated region of the CpG island flanking the bi-directional *BRCA1*–*NBR2* promoter. Positions are indicated from the *BRCA1* transcription start site. **(C)** *MiR-19a* can interact with *NBR2* and induce its degradation. **(D)** Some exogenous factors, such as hypoxia/energy stress in TME and antitumor drugs, can induce the expression of *NBR2*.

The analysis of the DNA sequence homology in the region that encompasses both the bi-directional promoter and the *BRCA1* promoter β (nucleotide positions 1191 to 2052) indicates a role for the CCAAT element in the coordinated activation of both *BRCA1* and *NBR2* genes (62). Other elements that can modulate the transcription of *BRCA1* and *NBR2* genes have also been identified (63–65). In the intergenic region between *BRCA1* and *NBR2* genes, a minimal 56-bp *Eco*RI–*Hae*III fragment has been delineated to act as a bi-directional promoter and it induces transcription in the *NBR2* gene direction 2–4-fold higher than that in the *BRCA1* direction in all tested cell lines (including cervical carcinoma, colon carcinoma, and mammary carcinoma cells). Within this sequence, the potential binding sites for TFs of the E-twenty-six (ETS) family, Sp1 transcription factor (SP1) family, and cAMP-responsive element-binding protein (CREB) have been defined. In addition, a specific protein–DNA complex was identified when this 56-bp *Eco*RI–*Hae*III minimal promoter was incubated with nuclear extracts from cancer cells. Moreover, the 56-bp minimal promoter can be further divided into 38-bp *Eco*RI–*Msp*I and 18-bp *Msp*I–*Hae*III fragments, and tissue-specific factor binding to *Msp*I–*Hae*III is required for *BRCA1* transcription (65). Another 36-bp *BstN*I–*BseR*I fragment, which is 575-bp into the first intron of the *BRCA1* gene, exhibits non-tissue-specific transcriptional suppressor activity by interacting with specific nuclear proteins. However, this putative negative regulatory element (NRE) only blocks transcription in the *BRCA1* direction, although the promoter is shared by the divergently transcribed *NBR2* gene (63). For the *NBR2* gene, an 18-bp transcriptional repressive element, which resides 948-bp into its first intron, was recently identified. The interaction between nuclear proteins and this 18-bp *Hae*III–*Hae*III repressive element was confirmed by electrophoretic mobility shift assays (EMSAs), and functional suppression was conferred to the heterologous thymidine kinase promoter by this element. In addition, this repressive element had no effect on the *BRCA1* direction in the context of its native genomic organization (64). Therefore, a model of the *BRCA1*–*NBR2* bi-directional transcription unit is established, in which the minimal 56-bp DNA region functions to drive the transcription in both directions and the uni-directional transcription is controlled by distinct repressors binding to elements in the first intron of respective genes (**Figure 2A**).

Specific molecules that regulate *NBR2* transcription by interacting with corresponding regulatory elements have been identified. For instance, methyl-CpG binding domain protein 2 (MBD2) specifically binds to the methylated region of the repeated LTR12c element at the *BRCA1*–*NBR2* locus, which leads to the silencing of the *NBR2* gene (66). MBD2 is a member of the MBD protein family, which are key molecules that participate in the interpretation of DNA methylation, leading to gene silencing (67). In the study by Auriol E et al., MBD2 was found to bind to the methylated region of the CpG island flanking the bi-directional *BRCA1*–*NBR2* promoter in HeLa cells, but not the unmethylated region. Meanwhile, the methylated region of the *BRCA1* island was not bound by other MBD proteins, such as methyl-CpG binding protein 2 (MeCP2) and MBD1, implying that the binding of MBD2 is specific. The *BRCA1*–*NBR2* locus-bound MBD2 induced the specific methylation-dependent repression of *NBR2*, whereas it did not affect the transcription of *BRCA1* (**Figure 2B**) (66). In addition to the regulation of *NBR2* expression at the transcriptional level, the modulation of the expression of this lncRNA at the post-transcriptional level has also been identified. In acute liver failure (ALF), increased *miR-19a* expression is accompanied by decreased *NBR2* expression. *MiR-19a* can interact with *NBR2* and protein kinase AMP-activated catalytic subunit alpha 1 (*PRKAA1*) (the gene encodes adenosine 5′-monophosphate (AMP)-activated protein kinase (AMPK)), and down-regulate the levels of both *NBR2* and AMPK in injured hepatocytes, thereby inhibiting autophagy in hepatocytes (**Figure 2C**) (59).

In addition to these endogenous regulatory patterns, *NBR2* expression can also be mediated by exogenous factors such as TME conditions and antitumor drugs. TME is a complex network consisting of a variety of cell types and factors, which is essential for tumor progression (68). Hypoxia, which is a common feature of TME and critical for the evolution of malignant cells, can affect *BRCA1* and *NBR2* expression differently. Hypoxia results in histone modifications at the endogenous *BRCA1* promoter in human breast cells, thereby repressing *BRCA1* transcription, whereas it induces the activation of *NBR2* transcription from the bi-directional *BRCA1* promoter. Different regulatory elements drive the hypoxia-induced repression of *BRCA1* and the activation of *NBR2* in cancer cells. It has been reported that hypoxia-induced silencing of the 218-bp minimal promoter is responsible for *BRCA1* down-regulation, whereas elements that control *NBR2* expression are beyond this minimal promoter and remain to be determined (69, 70). In addition, glucose starvation in TME can also induce the expression of lncRNA *NBR2* in cancer cells through the liver kinase B1 (LKB1)–AMPK pathway, and the precise mechanism will be discussed in the next section (45). Several antitumor drugs, such as phenformin and curcumin, have been reported to induce *NBR2* expression in cancer, although the precise regulatory mechanisms remain unknown (**Figure 2D**) (71, 72). *NBR2* is involved in cancer progression based on its ectopic expression. Therefore, we place an emphasis on the *NBR2*-indued regulatory mechanisms in tumorigenesis in the following section.

## Functions of *NBR2* in Cancer Biology

In consideration of the high mutation/deletion rate of the *BRCA1* gene in human breast and ovarian cancers, the *NBR2* gene, which is proximal to the *BRCA1* gene, was initially presumed to be co-deleted/mutated with *BRCA1* in certain cancers. Thus, *NBR2* may also play a role in tumor suppression similar to *BRCA1*. However, *NBR2* was later confirmed to be a lncRNA, and its complicated roles in tumor biology are being revealed gradually (45, 51, 73).

### Dual Role of NBR2 in Regulating Glucose Metabolism

**Figure 3:**
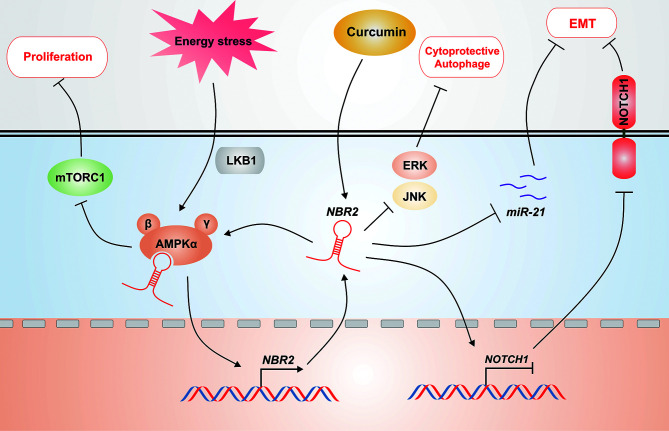
*NBR2* functions as a tumor suppressor. *NBR2* is responsible for the glucose metabolism in cancer dependent on the AMPK–mTOR pathway. Glucose starvation in TME induces the expression of *NBR2* depending on the LKB1–AMPK pathway. As feedback, stress-induced *NBR2* enhances the activity of AMPK kinase by directly interacting with its AMPKα subunit, thereby inhibiting the proliferation of cancer cells. Based on its regulation of the AMPK–mTOR pathway, curcumin-induced *NBR2* enhances the antitumor effects of curcumin by suppressing cancer proliferation and clone formation. In addition, *NBR2* attenuates EMT by blocking the NOTCH1 pathway and inhibiting *miR-21* expression. Moreover, *NBR2* impedes Beclin1-induced cytoprotective autophagy, thereby inhibiting cancer proliferation through ERK and JNK pathways.

The study by Liu X et al. identified the role of lincRNA *NBR2* in tumor suppression for the first time, and it was confirmed that energy stress-induced *NBR2* interacts with AMPK and potentiates AMPK activity under a condition of energy stress (45, 51, 73). Glucose deprivation in TME can subsequently result in increased cell apoptosis and decreased cell migration and invasion. However, to resist energy stress-induced apoptosis, cancer cells can adjust to the nutrient-limited environment and develop compensatory ways through metabolic reprogramming. Different from normal cells, cancer cells exhibit increased glucose uptake, aerobic glycolysis, enhanced glutamine uptake and glutaminolysis, and changes in lipid metabolism (60, 74). The findings of Liu X et al. were derived from the identification and investigation of energy stress-induced lncRNAs. To verify the lncRNA expression profile induced by energy stress, RNA sequencing was conducted in carcinoma cells cultured in medium with or without glucose. *NBR2* was identified as one of the glucose starvation-induced lincRNAs in the subsequent computational analysis, and its expression induced by energy stress was partly dependent on the LKB1–AMPK pathway. Furthermore, *NBR2* was demonstrated to regulate AMPK activity *via* a direct interaction (45). AMPK is a heterotrimeric complex containing a catalytic α subunit and two regulatory β and γ subunits (75). Stress-induced *NBR2* interacted with AMPKα of the AMPK complex through its first exon and enhanced the activity of AMPK kinase, which is parallel to LKB1-induced AMPK activation. Notably, *NBR2* was not required for initial energy stress-induced AMPK activation. Therefore, the feedback mechanism responsible for *NBR2*–AMPK regulation in both breast and kidney cancer cells under glucose-starvation conditions was revealed. AMPK is a crucial checkpoint of metabolism. Under conditions of energy stress, AMPK signaling is activated to facilitate catabolic processes (such as autophagy, fatty acid oxidation, glycolysis) and suppress anabolic processes (such as sterol/lipid/protein synthesis), thereby leading to restored energy balance. Mammalian target of rapamycin complex 1 (mTORC1)-induced protein synthesis and cell growth are major anabolic processes inhibited by AMPK in response to energy stress (76, 77), and energy stress-induced AMPK can also promote autophagy by directly activating the Unc-51 like autophagy activating kinase 1 (ULK1) through the phosphorylation of serine (Ser) 317 and Ser 777 (78). Therefore, the AMPK pathway functions to suppress cancers, as anabolic processes are necessary for tumor progression. Consistent with this finding, *NBR2* led to decreased cell cycle progression, but increased autophagy, which down-regulated apoptosis under energy stress and inhibited the progression of breast and kidney cancers, suggesting that *NBR2* is a tumor suppressor that regulates AMPK activity. Accordingly, the *NBR2* level was down-regulated in breast and kidney cancer patients and was negatively correlated with poor prognosis (45, 51, 73). In colorectal cancer (CRC), the *NBR2*–AMPK pathway is involved in curcumin-mediated CRC progression. It has been reported that glucose starvation induces the expression of *NBR2* in CRC cells, at least partly through an AMPK-dependent manner. *NBR2* overexpression results in the activation of the AMPK–mammalian target of rapamycin kinase (mTOR) pathway under glucose-starvation conditions, thereby affecting CRC progression (72). Curcumin, a compound isolated from the rhizomes of *Curcuma longa*, has been approved for the intervention of human cancers, including CRC (79). Curcumin shows a synergetic effect with glucose starvation in regulating the AMPK–mTOR pathway. Similar to glucose deficiency, curcumin induces *NBR2* expression in CRC cells. The increased *NBR2* enhances the curcumin-mediated suppression of the proliferation and clone formation of CRC cells through the activation of the AMPK–mTOR pathway, providing new insights for CRC therapy (**Figure 3**) (72).

**Figure 4:**
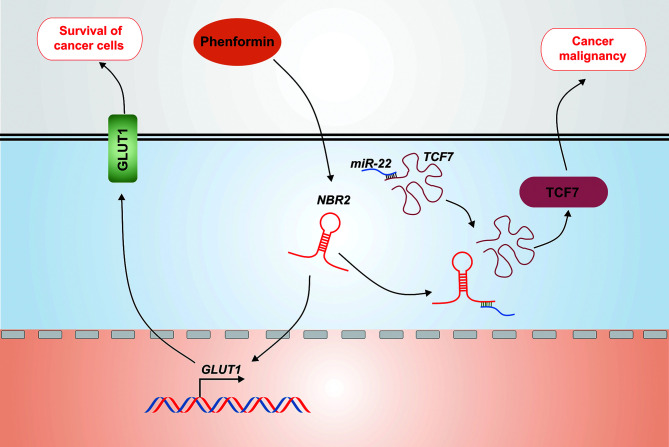
Opposite roles of *NBR2* in cancer. *NBR2* functions as an adaptive responser that maintains cell survival in response to phenformin treatment by facilitating *GLUT1* expression. In addition, *NBR2* in the cytoplasm sponges *miR-22* under conditions of glucose starvation, thereby counteracting *miR-22*-induced TCF7 repression and aggravating the malignancy of hepatoblastoma cells.

In addition to affecting the activation of the AMPK pathway, lncRNA *NBR2* can mediate the glucose metabolism of cancer cells by other mechanisms. In breast and kidney cancer cells, *NBR2* regulates cancer cell sensitivity to phenformin through glucose transporter 1 (GLUT1). Phenformin is an inhibitor of mitochondrial respiratory chain complex I, and its antitumor effect is more potent than that of metformin. Phenformin induces *NBR2* expression in breast and kidney cancer cells, and *NBR2* deficiency renders cancer cells more sensitive to phenformin-induced apoptosis. Thus, *NBR2* expression may function as an adaptive response to maintain cell survival after phenformin treatment. It is well established that phenformin enhances the activation of AMPK and the inactivation of mTORC1, and the inhibition of the AMPK pathway renders cancer cells more sensitive to phenformin-induced cell death. However, even if *NBR2* promotes energy-induced AMPK activation through direct interaction, the interaction of *NBR2* with AMPK is not influenced by phenformin, and phenformin induces AMPK activation independent of *NBR2*. On the other hand, *NBR2* deficiency suppresses glucose uptake through the inhibition of phenformin-induced GLUT1 expression, which is the glucose transporter with the most relevance to cancer biology, as it is overexpressed in many human cancers. It has been observed that GLUT1 deficiency sensitizes cancer cells to phenformin-induced cell death, whereas GLUT1 restoration in *NBR2*-deficient cells rescues the increased cell death after phenformin treatment. This finding identifies a new mechanism of *NBR2* modulation of glucose metabolism in cancer cells, suggesting that *NBR2* may predict the biguanide treatment response in cancer patients (71, 80). Furthermore, it reveals the opposite role of lncRNA *NBR2* in cancer development, which is contrary to the initial view that *NBR2* functions as a tumor suppressor. Consistently, *NBR2* has recently been identified to aggravate hepatoblastoma cell malignancy. *NBR2* expression is significantly increased in hepatoblastoma tissues, and glucose starvation is required for this up-regulation. The deficiency of *NBR2* suppresses the invasion, migration, and viability of hepatoblastoma cells cultured under normal conditions, and facilitates cell apoptosis under glucose starvation. As the most common pediatric liver malignancy, hepatoblastoma is believed to be a Wnt–β-catenin-activated malignant tumor, with frequent mutations in the catenin beta 1 (*CTNNB1*) gene that encodes β-catenin. Within mammalian cells, the activation of Wnt–β-catenin pathway leads to β-catenin accumulation in the cytoplasm and its translocation into the nucleus to facilitate the transcriptional activation of the T-cell factor (TCF) family (81–83). *TCF7* has been reported to be co-expressed with *NBR2*, and *NBR2* deficiency leads to decreased *TCF7* expression in hepatoblastoma cells, along with the down-regulation of the TCF7 protein that is involved in cell cycle progression, glucose entrapment, and epithelial-mesenchymal transition (EMT). The main regulatory mechanism of lncRNAs in the cytoplasm is to act as competing endogenous RNAs (ceRNAs) to sponge miRNAs. TCF7 has been defined as the target molecule of *miR-22* and *NBR2*, which is localized in both the cytoplasm and nucleus, aggravates hepatoblastoma cell malignancy by sponging *miR-22* under conditions of glucose starvation, thereby counteracting *miR-22*-induced TCF7 repression (**Figure 4**) (60). The above studies reveal the double-edged sword role of lncRNA *NBR2* in regulating the glucose metabolism of different cancers, further emphasizing its importance in maintaining the energy balance.

### NBR2 Suppresses EMT in Cancer

EMT is a process that is critical for wound healing, embryogenesis, and malignancy. During EMT, cell–extracellular matrix and cell–cell interactions are reprogrammed, which leads to the separation of epithelial cells from adjacent cells and the basement membrane, and new transcriptional events are induced to promote the mesenchymal fate. In cancer progression, EMT contributes to tumor initiation, metastasis, and therapeutic resistance, which is regulated by crucial TFs such as zinc-finger E-box-binding (ZEB) TFs and SNAIL. Both transcriptional reprogramming and non-transcriptional changes during EMT are induced by pathways responding to extracellular molecules (84, 85). Currently, lncRNAs are emerging as crucial regulators of EMT that can determine cancer progression (86). *NBR2*, an lincRNA that was initially identified as a cancer suppressor, is involved in the regulation of EMT in different cancers (**Figure 3**) (87–90).

In non-small cell lung cancer (NSCLC), the *NBR2* level is low in tumor tissues and is correlated with tumor size and prognosis. *NBR2* overexpression inhibits the proliferation, invasion, and migration of NSCLC cells. A mechanistic study has revealed that *NBR2* inhibits EMT in NSCLC by suppressing the notch receptor 1 (NOTCH1) pathway (87). During the development of osteosarcoma, cancer patients with low *NBR2* expression exhibit a shorter overall survival compared to those with high *NBR2* expression. *NBR2* inhibits the proliferation, invasion, and migration of osteosarcoma cells, but has no effect on cell apoptosis, thereby delaying tumor growth. *NBR2* has also been identified to function as an EMT suppressor by regulating NOTCH1 at both transcriptional and post-transcriptional levels in osteosarcoma (89). Similarly, the *NBR2* level is decreased in thyroid cancer (TC) tissues and cells, and it is associated with the histologic subtypes of TC. *NBR2* overexpression significantly suppresses TC proliferation, clonogenicity, and invasion as well as tumor growth *in vivo*, whereas *NBR2* knockdown has the opposite effects. Mechanistic studies have revealed that *NBR2* inhibits GLUT1 expression and EMT, but promotes AMPK and acetyl-CoA carboxylase (ACC) activation in TC cells, thereby reducing the malignancy of TC and acting as a tumor suppressor (90, 91). In colorectal cancer, the low *NBR2* level has been reported in tumor tissues, and decreased *NBR2* expression is associated with the progression of clinical stages. The expression of *miR-21*, which is a cancer promoter, is increased in colorectal cancer tissues and inversely correlated with the *NBR2* level. Moreover, *NBR2* overexpression inhibits *miR-21* expression in colorectal cancer cells and attenuates colorectal cancer cell migration and invasion, whereas *miR-21* has no influence on *NBR2* expression (88). These findings revealing the role of *NBR2* in EMT further confirm that *NBR2* may be a potential target for cancer diagnosis and treatment.

### NBR2 Regulates the Autophagy of Cancer Cells

Autophagy is a conserved catabolic process. It is initiated by the formation of autophagosomes, which encapsulate the cytoplasm and organelles, and then form autolysosomes by fusing with lysosomes, thereby leading to the degradation of the contents contained within the vesicles (92). Autophagy mediates the proliferation and apoptosis of liver cells in different contexts, and it has been confirmed that protective autophagy is the main cause of cancer survival in an adverse environment (93, 94). In addition to affecting the metabolism and EMT, *NBR2* can also inhibit tumorigenesis by regulating autophagy. It has been demonstrated that in hepatocellular carcinoma (HCC) patients at advanced clinical stages, the overall survival of cases with low *NBR2* expression is significantly worse compared to those with high *NBR2* expression. As such, *NBR2* acts as a negative regulator of HCC, as it inhibits cancer proliferation, invasion, and migration. Cell death is a complicated process, for which autophagy, as well as apoptosis, is essential. *NBR2* regulates autophagy, but not apoptosis, of HCC cells. Basal autophagy suppresses tumor progression by maintaining cellular homeostasis. However, protective autophagy enhances the survival of cancer cells to facilitate tumor development in TME. Therefore, an inhibition of autophagy may be an ideal approach for cancer treatment. In HCC, *NBR2* attenuates Beclin1-induced cytoprotective autophagy to inhibit cancer proliferation through the extracellular regulated protein kinase (ERK) and c-Jun N-terminal kinase (JNK) pathways, which provides novel insights on a treatment strategy for HCC (95, 96).

## Clinical Applications of *NBR2* in Cancer

Similar to *BRCA1*, the potential application of *NBR2* as a cancer biomarker was initially revealed in breast cancer, as the expression of *NBR2* decreased in primary cancer cells derived from human breast cancer tissues (61). Subsequent studies further confirmed this finding and showed that low *NBR2* expression correlates with poor clinical outcomes in breast and ovarian cancer patients (45, 97). Furthermore, a common breast cancer risk loci, rs9911630, is identified to be the most strongly expression-associated genotyped single nucleotide polymorphism (SNP) that affects the expression of *BRCA1* and *NBR2* in the Tunisian population, but whether this SNP is responsible for *NBR2* expression in other populations is still not clear (98). Germline mutations in the coding region of the *BRCA1* gene are responsible for familial breast and ovarian cancers (51–53). Different from *BRCA1*, there is no mutation in the *NBR2* gene (46). However, it has been demonstrated that the deletion of the *NBR2* gene may be closely related to the susceptibility of breast and ovarian cancers in different populations. For example, germline *BRCA1* promoter deletions have been confirmed in familial breast cancer patients from the United Kingdom and Australia. The breakpoints for this deletion are in *BRCA1* intron 2 and between *NBR2* and *BRCA1P1* exon 2, suggesting that this deletion takes place through a novel mechanism involving the recombination of *BRCA1*:*BRCA1P1* (99). In a French breast–ovarian cancer family, a novel rare 161-kb deletion in the region extending from the *NBR1* gene to the *BRCA1* gene was identified. This deletion encompassed *NBR1*, *BRCA1P1*, *NBR2*, and *BRCA1* genes, and it started from the Alu Y sequence of *NBR1* intron 18 and ended at the Alu Sc sequence of *BRCA1* intron 22. The hemizygosity of the four genes showed no specific phenotype (100). For sporadic breast cancer, a *de novo* complete *BRCA1* gene deletion, which includes Rho family GTPase 2 (*RND2*), *BRCA1P1*, *BRCA1*, and *NBR2* complete genes, has been reported in a Spanish woman with early bilateral breast cancer, supporting the large genomic rearrangement screening of *BRCA* genes in young breast cancer patients without a family history, as well as in hereditary breast and ovarian cancer families previously tested negative for other variations (101). In an Italian woman diagnosed with high-grade serous ovarian carcinoma, the deletion of a 137.8-kb region, encompassing the first six exons of the *BRCA1* gene and the full length of *NBR2*, *BRCA1P1*, *NBR1*, and transmembrane protein 106A (*TMEM106a*) genes, was detected (102). In addition to breast and ovarian cancers, dysregulated *NBR2* expression and function have also been observed in some other solid tumors (**Table 1**). Consistently, the *NBR2* level was associated with the progression of these cancers (72, 80, 89, 90, 95, 97). For instance, the *NBR2* level in advanced HCC patients is correlated with overall survival (95). These studies suggest that *NBR2* is a potential biomarker for monitoring cancer development.

**Table 1:** Clinical applications of *NBR2* in cancer.

Cancer type	Role of *NBR2*	Outcome	Potentialapplication	Refs
Glioma	Oncogene	Viability ↑ Proliferation ↑ Migration ↑ Invasion ↑	Diagnosis /Prognosis /Therapy	(103)
Hepatoblastoma	Oncogene	Viability ↑ Proliferation ↑ Migration ↑ Invasion ↑	Diagnosis /Therapy	(60)
Hepatocellular carcinoma	Tumor suppressor	Proliferation ↓ Migration ↓ Invasion ↓ Autophagy ↓	Diagnosis /Prognosis /Therapy	(95)
Thyroid cancer	Tumor suppressor	Proliferation ↓ Clonogenicity ↓ Invasion ↓ Wound healing ↓ Apoptosis ↑	Diagnosis /Therapy	(90)
Non-small-cell lung cancer	Tumor suppressor	Viability ↓ Migration ↓	Diagnosis /Prognosis /Therapy	(87)
Colorectal cancer	Tumor suppressor	Invasion ↓ Migration ↓ Proliferation ↓ Curcumin sensitivity↑	Diagnosis /Prognosis /Therapy	(88)
Ovarian cancer	Tumor suppressor	–	Prognosis	(97)
Osteosarcoma	Tumor suppressor	Proliferation ↓ Migration ↓ Invasion ↓	Diagnosis /Prognosis /Therapy	(89)
Breast & Renal cancer	Tumor suppressor	Proliferation ↓ Phenformin sensitivity ↓	Diagnosis /Prognosis /Therapy	(45, 99–102)

Roles of NBR2 in different cancer types and its potential clinical applications. “-” represents “not identified”.

*NBR2* is also a promising therapeutic target, as *NBR2* is involved in regulating the proliferation, migration, and survival of different cancer cells (45, 87, 90, 95). Moreover, *NBR2* affects cancer cell sensitivity to antitumor drugs, as *NBR2* expression is related to drug resistance (104). As mentioned above, *NBR2* deficiency renders cancer cells more sensitive to phenformin through the inhibition of *GLUT1* expression, suggesting that the *NBR2*–GLUT1 axis may serve as an adaptive response for phenformin treatment (71, 80). In CRC, increased *NBR2* expression enhances the antitumor effect of curcumin by activating the AMPK–mTOR pathway (72). Therefore, it is conceivable to expand the utilization of *NBR2* in antitumor therapies.

## Challenges and Future Directions

Despite considerable progress in the understanding of *NBR2* function, significant obstacles remain to be overcome for better realizing the role of *NBR2* in cancer. One challenge is the lack of a high-resolution map of *NBR2*’s interactions with its partners. LncRNA regulation is associated with its location in cells, and *NBR2* has been indicated to be localized in both the cytoplasm and nucleus, prompting the diversity of *NBR2* regulatory mechanisms (19, 45). *NBR2* in the cytoplasm can promote AMPK activity by interacting with AMPKα, thereby regulating the proliferation, apoptosis, and autophagy of cancer cells (45). However, it is still unclear whether the *NBR2*–AMPK complex encompasses other molecules or whether the interaction of *NBR2* with other proteins has the same effect. Therefore, the identification of molecules interacting with *NBR2* will be helpful for defining the functions of *NBR2*. Moreover, *NBR2* dysregulation can regulate the expression of cancer-associated genes, such as *NOTCH1* and *GLUT1*, suggesting that *NBR2* can affect the activation of gene transcription (71, 87). However, the precise mechanism of *NBR2* in the regulation of gene expression at the transcriptional level still needs to be investigated.

The dual role of *NBR2* in cancer biology is another challenge. *NBR2* was initially identified as a tumor suppressor similar to *BRCA1*, and plenty of evidence has verified this perspective (45). However, an opposite role of *NBR2* in cancer progression has also been revealed. It has been reported that the *NBR2* level is significantly increased in hepatoblastoma tissues, where it aggravates hepatoblastoma cell malignancy under conditions of glucose starvation through the *miR-22*–TCF7 axis (60). Furthermore, *NBR2* induces the resistance of cancer cells to phenformin treatment (71, 80). A recent study has also confirmed that *NBR2* promotes the proliferation of glioma cells by inhibiting *p15* expression (103). The adverse effects of *NBR2* may result from the type, stage, or genetic context of these cancers, underlining that antitumor therapeutic strategies targeting *NBR2* should be applied modestly.

Lastly, based on its mutation and dysregulated expression, the *BRCA1* gene is one of the most important genes for the susceptibility of breast and ovarian cancers, and its clinical application in cancer diagnosis has been reported (50, 105). The potential application of the *NBR2* gene, which shares a bi-directional promoter with the *BRCA1* gene, has also been revealed in cancer diagnosis (95). The deletion of the *NBR2* gene has been reported to be associated with the susceptibility of breast and ovarian cancers in different populations. However, these deletions, which usually contain *BRCA1*, *NBR2*, *BRCA1P1*, and *NBR1* genes, are not restricted to the *NBR2* gene, and further studies are needed to confirm whether a specific deletion of the *NBR2* gene determines cancer susceptibility.

## Conclusions

*NBR2* has emerged as a crucial regulator of cancer biology, and a promising diagnostic and therapeutic target. Further investigations underlying the *NBR2* regulatory mechanisms in cancer are required for the materialization of its clinical application. Finally, these studies will improve our understanding of the roles of lncRNA in cancer through the investigation of *NBR2*.

## Author Contributions

TW, FY, and XT conceived the presented ideas and researched the background of the study. TW, ZL, and HS prepared the figures and tables. TW, LY, and XT wrote the manuscript. All authors contributed to the article and approved the submitted version.

## Funding

This work was supported by the National Natural Science Foundation of China (Grant No. 81802855), the Natural Science Foundation of Jiangsu Province (Grant No. BK20180123), the Jiangsu Postdoctoral Research Foundation (Grant No. 2018K253C), and the China Postdoctoral Science Foundation (Grant No. 2018ZM642225).

## Conflict of Interest

The authors declare that the research was conducted in the absence of any commercial or financial relationships that could be construed as a potential conflict of interest.

## Publisher’s Note

All claims expressed in this article are solely those of the authors and do not necessarily represent those of their affiliated organizations, or those of the publisher, the editors and the reviewers. Any product that may be evaluated in this article, or claim that may be made by its manufacturer, is not guaranteed or endorsed by the publisher.
